# Risk factors that may be driving the emergence of drug resistance in tuberculosis patients treated in Yangon, Myanmar

**DOI:** 10.1371/journal.pone.0177999

**Published:** 2017-06-14

**Authors:** Mishal S. Khan, Coll Hutchison, Richard J. Coker

**Affiliations:** 1 Communicable Diseases Policy Research Group, London School of Hygiene and Tropical Medicine, London, United Kingdom; 2 Saw Swee Hock School of Public Health, National University of Singapore, Singapore; 3 Faculty of Public Health, Mahidol University, Bangkok, Thailand; McGill University, CANADA

## Abstract

**Background:**

The majority of new tuberculosis cases emerging every year occur in low and middle-income countries where public health systems are often characterised by weak infrastructure and inadequate resources. This study investigates healthcare seeking behaviour, knowledge and treatment of tuberculosis patients in Myanmar—which is facing an acute drug-resistant tuberculosis epidemic—and identifies factors that may increase the risk of emergence of drug-resistant tuberculosis.

**Methods:**

We randomly selected adult smear-positive pulmonary tuberculosis patients diagnosed between September 2014 and March 2015 at ten public township health centres in Yangon, the largest city in Myanmar. Data on patients’ healthcare seeking behaviour, treatment at the township health centres, co-morbidities and knowledge was collected through patient interviews and extraction from hospital records. A retrospective descriptive cross-sectional analysis was conducted.

**Results:**

Of 404 TB patients selected to participate in the study, 11 had died since diagnosis, resulting in 393 patients being included in the final analysis. Results indicate that a high proportion of patients (16%; 95% CI = 13–20) did not have a treatment supporter assigned to improve adherence to medication, with men being more likely to have no treatment supporter assigned. Use of private healthcare providers was very common; 59% (54–64) and 30.3% (25.9–35.0) of patients reported first seeking care at private clinics and pharmacies respectively. We found that 8% (6–11) of tuberculosis patients had confirmed diabetes. Most patients had some knowledge about tuberculosis transmission and the consequences of missing treatment. However, 5% (3–8) stated that they miss taking tuberculosis medicines at least weekly, and patients with no knowledge of consequences of missing treatment were more likely to miss doses.

**Conclusions:**

This study analysed healthcare seeking behaviour and treatment related practices of tuberculosis patients being managed under operational conditions in a fragile health system. Findings indicate that ensuring that treatment adherence support is arranged for all patients, monitoring of response to treatment among the high proportion of tuberculosis patients with diabetes and engagement with private healthcare providers could be strategies addressed to reduce the risk of emergence of drug-resistant tuberculosis.

## Background

Despite decades of investment, tuberculosis (TB) is proving to be challenging to control for numerous reasons: lack of timely access to quality diagnostic and treatment services for vulnerable populations, a long (minimum six month) duration of treatment which places a great burden on patients and the health system, and dominance of private healthcare providers that often do not follow TB management guidelines in numerous high TB burden countries [1]. These factors have contributed to the emergence and spread of drug-resistant TB, which is threatening to reverse progress made in TB control in recent decades; globally, 21% (15–28%) of previously treated cases and 4% (3–5%) of new cases are estimated to have TB that is resistant to at least the two major anti-tuberculosis drugs, isoniazid and rifampicin [2].

The majority of the 480,000 new MDR-TB cases and approximately 10 million new TB cases emerging every year occur in low and middle-income countries. Health systems in these countries are often characterised by weak infrastructure and inadequate resources (referred to as fragile health systems) and they are therefore poorly equipped to cope with the complexities and expenses of diagnosing and managing the disease [2]. Unfortunately, countries that are least able to cope with a high MDR-TB burden are often those where the health system struggles to effectively treat all TB patients, driving the emergence of drug resistance [3, 4].

Myanmar is a low-income country whose fragile health system—which is receiving external support for human resource capacity building, medical equipment and programme implementation—has to cope with very high TB and MDR-TB rates [5]. The TB prevalence rate is 525 cases per 100,000 population—more than four times higher than the global average. An estimated 180,000 new TB cases emerge every year [5]. Among newly diagnosed cases, it is estimated that 5% of previously untreated and 27% of previously treated patients have MDR-TB; this is considerably higher than other countries in South East Asia, such as Cambodia (1.4% and 11% respectively) and Thailand (2% and 19% respectively) [2].

An understanding of patient behaviour before and during TB treatment in the context of a fragile health system is important as it may indicate factors associated with the potential generation of MDR-TB [6]. This study investigates healthcare seeking behaviour, knowledge and treatment of tuberculosis patients under operational conditions in Myanmar’s resource-constrained public health system.

## Methods

### Study setting

Health services in Yangon, which is the country’s main urban centre with a population of 7.4 million, are provided by a combination of the public and private sector. Some private healthcare providers, such as the Myanmar Medical Association (MMA) and Population Services International (PSI) linked SUN clinics, are affiliated with the National Tuberculosis Programme (NTP) to provide standardised TB services. The majority of private healthcare providers are, however, unregulated by any government bodies and include small clinics, hospitals, pharmacies and informal drug sellers. The national Ministry of Health is responsible for the management of the public sector services and the NTP oversees TB control activities. TB control activities are implemented through Yangon’s Township Health Departments (THDs), which function as the main health facilities providing primary health care services and as the first point of accessing services for TB diagnosis, case registration and treatment provision. These services are provided without charge to patients. Our study was conducted in ten THDs across Yangon, identified by the NTP as having a particularly high MDR-TB burden (defined as having 2–4 new cases diagnosed per month). These THDs were located in the following townships: Hlaing, Hlaing Thar Yar, Insein, Mayangone, Mingaladon, North Dagon, North Okkalapa, Shwe Pyi Thar, South Okkalapa and Thingangyun.

### Participants

We randomly selected TB patients for inclusion rather than including all TB cases because the number of new cases diagnosed every month was very large, and we wanted to recruit patients over a minimum six-month period to obtain a representative sample. Random selection was weighted according to the MDR-TB burden in each study township, such that a proportionally larger number of TB patients would be selected from townships with higher MDR-TB patient numbers. Our exclusion criteria were: age <18 years; known pregnancy at time of data collection; residence outside Yangon or in Yangon <3 months; and extrapulmonary TB only.

### Data collection, management and analysis

After confirming eligibility and willingness to participate we retrospectively collected data from TB patients three months after they had been diagnosed. A three month period post diagnosis was selected to achieve a balance between optimal recall of behaviour in the period prior to diagnosis and to allow information about adherence to treatment in the initial phase of treatment to be collected. A data collector fluent in the local language and trained to administer the questionnaire was assigned to each township. Using Caminero’s classification of risk factors for MDR-TB [4] as a starting point, we designed and piloted data collection tools for capturing information on patient and health system variables. Two structured questionnaires were filled out for each participant. The first extracted data from townships labs, TB registers at the township health centres and patients’ individual treatment cards. The second questionnaire was based on primary data collected during face-to-face interviews with patients at their homes.

Data was collected using paper-based questionnaires, checked manually by the study supervisor and then entered into Epidata (Version 3.1, The EpiData Association, Odense,8.

Denmark). The Epidata file had built-in checks to alert the data entry manager if out of range values were entered. Blinded double entry was conducted by the study supervisor on a monthly basis for 20% of all questionnaires (randomly selected). If any errors were identified, all questionnaires from the same data collector were checked and the data collector re-trained on the relevant questions if required.

Raw data was transferred to Stata (version 10, Stata Corporation, College Station, TX) for management and statistical analysis. We conducted a descriptive analysis and report numbers and percentages for the categorical variables we analysed. Where indicated in the results section, we conducted additional analysis of association between risk factors and specific patient characteristics using the Pearson chi-squared test.

### Ethics

Ethical clearance was obtained from LSHTM Research Ethics Committee, the FHI 360 Protection of Human Subjects Committee and the Myanmar Ministry of Health. Written informed consent was sought from all participants.

## Results

Between Sept 2014 and March 2015, 1691 new eligible smear-positive pulmonary TB patients were diagnosed at the ten study THDs. We excluded 19 patients owing to missing information on age and sex, leaving a sampling frame of 1672. We randomly selected 404 TB patients to participate in the study, of which 11 had died since diagnosis, resulting in 393 patients being included in the final analysis (S1 dataset).

Demographic and socioeconomic characteristics of the study population are summarised in Table 1. Health system related factors related to healthcare seeking behaviour and treatment support are summarised in Table 2. Our results indicate high use of (unregulated) private healthcare providers that do not offer free, standardised diagnosis and treatment for TB; in terms of actual healthcare seeking behaviour reported, 67.2% (62.4–71.7) of TB patients visited a private allopathic healthcare provider in the past two years. In contrast, reported use of non-allopathic healthcare providers (traditional healers) in the past two years was relatively low in Yangon at 4.1% (2.5–6.6) in the past two years. Our analysis of stated preferences for healthcare seeking revealed that over 90% of TB patients’ first choice is to seek care at a private health facility. Among private health facilities chosen for the first healthcare seeking visit, clinics (59.3% [54.3–64.1]) and pharmacies (30.3% [25.9–35.0]) were the two most commonly cited. Only 4.6% (2.9–7.2) of patients stated that a government facility would be their first choice. Once TB patients had started treatment through the NTP, less than 7% continued to seek care for TB symptoms at a private hospital, clinic or pharmacy.

**Table 1:** Demographic and socioeconomic characteristics of TB patients included in the study. * = private healthcare providers working in partnership with the National TB program

Characteristic	Number of Patients (N = 393)	Proportion of Patients (95% Confidence Intervals)
**Age**		
18–24	52	13.2 (10.2–17)
25–34	99	25.2 (21.1–29.7)
35–44	95	24.2 (20.2–28.7)
45–54	70	17.8 (14.3–21.9)
55–64	45	11.5 (8.6–15.0)
Over 65	32	8.1 (5.8–11.3)
**Female Sex**	125	31.8 (27.4–36.6)
**Ethnic group**		
Bamar	345	87.8 (84.1–90.7)
Mixed	9	2.3 (1.2–4.4)
Ethnic minority	39	9.9 (7.3–13.3)
**Religion**		
Buddhist	37	94.1 (91.3–96.1)
Christian	7	1.8 (0.8–3.7)
Muslim	14	3.6 (2.1–5.9)
Hindu	2	0.5 (0.1–2.0)
**Occupation**		
Dependent	81	20.6 (16.9–24.9)
Daily wage earner	41	10.4 (7.8–13.9)
Self or privately employed	171	43.5 (38.7–48.5)
Government employee	23	5.9 (3.9–8.7)
Unemployed	62	15.8 (12.5–19.7)
Retired/Other	15	3.8 (2.3–6.2)
**Education level**		
None or less than primary	77	19.6 (15.9–23.8)
Only primary completed	121	30.8 (26.4–35.6)
Only middle school completed	124	31.5 (27.1–36.3)
High school completed or higher	71	18.1 (14.6–22.2)
**Healthcare provider used for TB treatment**		
National TB Programme	314	79.9 (75.6–83.6)
Myanmar Medical Association *	32	8.1 (5.8–11.3)
Sun Clinic*	45	11.5 (8.6–15.0)
Private hospital	2	0.5 (0.1–2.0)

**Table 2:** Healthcare seeking behaviour and treatment support.

Variable	Number of Patients (N = 393)	Proportion of Patients (95% Confidence Intervals)
**Preferred (first) health care provider to visit when sick**		
Private clinic / hospital	233	59.3 (54.3–64.1)
Government run clinic / hospital	18	4.6 (2.9–7.2)
Private pharmacy	119	30.3 (25.9–35.0)
Informal drug seller	11	2.8 (1.6–5.0)
Informal care / traditional healer	2	0.5 (0.1–2.0)
Nobody	10	2.5 (1.4–4.7)
**Actual visits to a private doctor in the past 2 years**		
No	129	32.8 (28.3–37.6)
Yes	264	67.2 (62.4–71.7)
**Actual visits to a traditional healer in the past 2 years**		
No	377	95.9 (93.4–97.5)
Yes	16	4.1 (2.5–6.6)
**Visits to additional allopathic healthcare provider after TB diagnosis by NTP**		
No	283	72.0 (67.3–76.2)
MMA (NTP affiliated)	36	9.1 (6.7–12.5)
PSI / Sun clinic (NTP affiliated)	46	11.7 (8.9–15.3)
Private clinic	18	4.6 (2.9–7.2)
Private Hospital	6	1.5 (0.7–3.4)
Pharmacy	2	0.5 (0.1–2.0)
Other	1	0.3 (0–1.8)
Missing	1	0.3 (0–1.8)
**Visits to traditional healer after TB diagnosis by NTP**		
No	383	97.5 (95.3–98.6)
Yes	10	2.5 (1.4–4.7)
**TB treatment supporter assigned by health facility staff**		
None assigned	63	16.0 (12.7–20.0)
Household member	309	78.6 (74.3–82.4)
Non-household family member	17	4.4 (2.7–6.9)
Other	4	1.0 (0.4–2.7)

While health facilities allocated a family member to support treatment for the majority of TB patients (78.6% [74.3–82.4]), 16% (12.7–20.0) reported that they did not have treatment supporters assigned to monitor and support their adherence to TB medication. Further analysis indicated that male patients were less likely to be assigned a treatment supporter, with 18.7% (14.0–23.3) of men and 10.4% (5.0–15.8) of women (p = 0.038) being allocated no treatment supporter by the health facility. Other factors such as the patient’s age, ethnicity and marital status were not found to be associated with assignment of a treatment supporter (not shown in tables).

As summarised in Table 3, most patients (75.1% [70.5–79.1]) had some knowledge about how TB is transmitted and almost 95% were aware of some negative consequences of missing TB treatment. Several questions were asked to estimate adherence to treatment, all of which indicated that approximately 5% of TB patients regularly miss taking drugs. We found that 4.6% (2.9–7.2) of patients stated that they had missed taking treatment in the past 4 days, and similarly 4.8% (3.1–7.5) stated that they miss taking TB medicines weekly or more often. Over the past month, 6.7% (4.5–9.6) recalled that missing at least one dose of TB medication. There was a strong association between knowledge of consequences of missing treatment and missing treatment more than weekly (not shown in tables); 23.8% (5.6–42.2) of patients who had no knowledge of consequences of poor adherence to TB treatment reported missing treatment more than weekly, as compared to 3% (0–5.5) of patients who knew about emergence of drug resistance and 4% (1.8–6.8) of patients who were aware that symptoms can worsen (p<0.0001).

**Table 3:** Knowledge and adherence to treatment.

Variable	Number of Patients (N = 393)	Proportion of Patients (95% Confidence Intervals)
**Knowledge of how TB is spread**		
No	98	24.9 (20.9–29.5)
Yes (airborne/coughing)	295	75.1 (70.5–79.1)
**Knowledge of impact of missed treatment**		
Nothing	21	5.3 (3.5–8.1)
Drug resistance	115	29.3 (25–34)
Symptoms get worse	230	58.5 (53.6–63.3)
Drug resistant and symptoms get worse	27	6.9 (4.7–9.8)
**Patients reported missing treatment in last 4 days**		
No	375	95.4 (92.8–97.1)
Yes	18	4.6 (2.9–7.2)
**Patients reported missing treatment in last month**		
No	366	93.1 (90.2–95.3)
Yes	26	6.6 (4.5–9.6)
Missing	1	0.3 (0–1.8)
**How often patient missed taking TB drugs (self reported)**		
Never	365	92.9 (89.9–95)
Less than once a week	9	2.3 (1.2–4.4)
More than once a week	19	4.8 (3.1–7.5)
**Reasons provided for missing TB drugs**		
Did not miss taking drugs	365	92.9 (89.9–95)
Forgot	3	0.7 (0.2–2.4)
Ran out / forgot to pick up	7	1.8 (0.8–3.7)
Side effects	5	1.3 (0.5–3)
Other	13	3.3 (1.9–5.6)

At the individual level (Table 4), there was a high prevalence of confirmed diabetes (7.9% [5.6–11.0]) among TB patients. We found that information on HIV status was incomplete as routine testing of all TB patients was not implemented at THDs.

**Table 4:** Factors influencing individual patients’ vulnerability.

Variable	Number of Patients (N = 393)	Proportion of Patients (95% Confidence Intervals)
**HIV status**		
Tested negative	251	63.9 (59–68.5)
Tested positive	13	3.3 (1.9–5.6)
Not tested	129	32.8 (28.3–37.6)
**Diabetes Status**		
Tested negative	298	75.8 (71.3–79.8)
Tested positive	31	7.9 (5.6–11.0)
Not tested	64	16.3 (12.9–20.3)
**Alcohol consumption**		
Never	184	46.8 (41.9–51.8)
Past drinker	175	44.5 (39.7–49.5)
Current drinker	34	8.7 (6.2–11.9)
**Smoking**		
Never	181	46.0 (41.2–51)
Past smoker	174	9.7 (7.1–13)
Current smoker	38	44.3 (39.4–49.2)

## Discussion

This study provides new information about healthcare seeking behaviour and management of TB patients under operational conditions in the context of a resource-limited setting, where investment and reform are ongoing to try and address the epidemiological challenge of high rates of TB and MDR-TB. We identified lack of treatment adherence support and high use of unregulated private healthcare providers as health system related factors potentially driving the generation of MDR-TB. In terms of patient-level factors, a substantial proportion of TB patients (7.9%) had confirmed diabetes, which is associated with a higher risk of poor TB treatment outcomes [7].

Despite being a central component of the World Health Organization’s TB control strategy, we found that 16% of patients had no treatment supporter. There is increasing evidence that direct observation of treatment at a health centre does not improve treatment outcomes and that a community based treatment supporter is as effective as, or superior to, a health-service based treatment supporter [8, 9]. In our study setting, in line with other settings with limited human resources for health service delivery, family members were commonly selected to act as treatment supporters, which reduces burden on the health system. However, our finding that a considerable proportion of patients, particularly men, had no treatment supporter assigned to them indicates that steps required to identify and coach a family or community member to act as a treatment supporter were not being taken consistently at the public health facilities in our study. Furthermore, while we sought to include HIV-coinfection in our analysis, we found that one third of TB patients did not have HIV test results recorded in township registers, and suggest that routine HIV testing should be considered for all TB patients. We acknowledge, however, that resource constraints within THDs may be important barriers to ensuring comprehensive coverage of HIV testing and treatment supporters.

Our study in Myanmar also provides new information about healthcare seeking behaviour of TB patients when the private sector is a dominant component of the health system [10]. In this context, the vast majority (over 90%) of TB patients reported first visiting a private healthcare facility rather than a public one, which is consistent with, but slightly higher than, rates reported in other Asian settings [11]. This is despite TB drugs and diagnostic tests being available without charge at public sector THDs, and no known waiting lists for first-line TB drugs. Use of allopathic health services, rather than traditional healers, was preferred by patients in this urban setting. Evidence from other studies indicates that TB patients prefer to use private health facilities owing to factors such as convenience, confidentiality and perceived quality of service, although quality of care is often suboptimal [12–14]. Specifically, for-profit healthcare providers operating in a setting with limited enforcements of laws to control service quality have been known to inappropriately dispense antibiotics increasing the risk of antibiotic resistance developing [15, 16]. While we did not investigate regulation of the private health sector or quality of TB care delivered by private healthcare providers as part of this study, other studies in Myanmar have shown that private allopathic clinics deviate from guidelines including observation of TB treatment [10], formal (written) monitoring of patient progress during treatment [17] and use of smear-microscopy for diagnosis in patients with symptoms consistent with TB [18].

A small but substantial proportion of patients reported frequently missing treatment; while overall patient knowledge of consequences of missing treatment was high, we found that the minority (5%) of patients who were not aware of any consequences of poor adherence to TB treatment were more likely to report missing treatment more than weekly. We recognise that a limitation of our study is that we only collected adherence data about the first three months of treatment, whereas adherence to treatment may worsen in later months. We also recognise the potential for under-reporting in data based on self-reported information about smoking, alcohol consumption and missed treatment.

## Conclusion

Our study identifies healthcare seeking and treatment adherence behaviour—as well as co-morbidities in TB patients—that should be investigated further as potential drivers of drug resistance. It is optimal from a patient and health system perspective to prevent the emergence of drug resistance, and this is recognised as the first step to controlling MDR-TB in high prevalence countries such as Myanmar [4]. The present study indicates potential strategies that may be considered to reduce of the risk of MDR-TB, such as introduction of processes at public health facilities that ensure consistency in assignment of treatment supporters to all TB patients and engagement with private healthcare providers to avoid inappropriate treatment of potential TB patients. Further studies evaluating the impact and cost-effectiveness of measures to prevent generation of drug resistance in patients treated for primary TB in resource-limited settings are warranted.

## Supporting information

S1 Dataset Dataset of TB patients used for the analysis. (XLSX)

## References

[1] Lönnroth K, Castro KG, Chakaya JM, Chauhan LS, Floyd K, Glaziou P, et al Tuberculosis control and elimination 2010–50: cure, care, and social development. The Lancet. 2010;375:1814–1829. 10.1016/S0140-6736(10)60483-7 20488524

[2] WHO. Global tuberculosis report 2016. Geneva, Switzerland: World Health Organization; 2016.

[3] Zager EM, McNerney R. Multidrug-resistant tuberculosis. BMC Infect Dis. 2008;8:10 doi: 10.1186/1471-2334-8-10 18221534 10.1186/1471-2334-8-10 PMC2241599

[4] Caminero JA. Multidrug-resistant tuberculosis: epidemiology, risk factors and case finding. Int J Tuberc Lung Dis. 2010;14:382–90. 20202293

[5] WHO. Multidrug-resistant tuberculosis in Myanmar Progress, Plans and Challenges. Yangon, The Republic of the Union of Myanmar: World Health Organisation, South-East Asia Region Office; 2012.

[6] Singh JA, Upshur R, Padayatchi N. XDR-TB in South Africa: no time for denial or complacency. PLoS Med. 2007;4:19. 10.1371/journal.pmed.0040050 PMC1779818 17253901

[7] Baker MA, Harries AD, Jeon CY, Hart JE, Kapur A, Lönnroth K, et al The impact of diabetes on tuberculosis treatment outcomes: A systematic review. BMC Med. 2011;9:81 doi: 10.1186/1741-7015-9-81 21722362 10.1186/1741-7015-9-81 PMC3155828

[8] Wright CM, Westerkamp L, Korver S, Dobler CC. Community-based directly observed therapy (DOT) versus clinic DOT for tuberculosis: a systematic review and meta-analysis of comparative effectiveness. BMC Infect Dis. 2015;15:210 doi: 10.1186/s12879-015-0945-5 25948059 10.1186/s12879-015-0945-5 PMC4436810

[9] Karumbi J, Garner P. Directly observed therapy for treating tuberculosis. Cochrane Database Syst Rev. 2015;:1–56. 10.1002/14651858.CD003343.pub4 PMC4460720 26022367

[10] Lönnroth K, Aung T, Maung W, Kluge H, Uplekar M. Social franchising of TB care through private GPs in Myanmar: an assessment of treatment results, access, equity and financial protection. Health Policy Plan. 2007;22:156–66. doi: 10.1093/heapol/czm007 17434870 10.1093/heapol/czm007

[11] Uplekar M, Pathania V, Raviglione M. Private practitioners and public health: weak links in tuberculosis control. The Lancet. 2001;358:912–6. 10.1016/S0140-6736(01)06076-7 11567729

[12] Hazarika I. Role of private sector in providing tuberculosis care: evidence from a population-based survey in India. J Glob Infect Dis. 2011;3:19–24. doi: 10.4103/0974-777X.77291 21572604 10.4103/0974-777X.77291 PMC3068573

[13] Uplekar MW, Shepard DS. Treatment of tuberculosis by private general practitioners in India. Tubercle. 1991;72:284–90. 1811360 10.1016/0041-3879(91)90055-w

[14] Zwi AB, Brugha R, Smith E. Private health care in developing countries: If it is to work, it must start from what users need. BMJ. 2001;323:463–4. 11532823 10.1136/bmj.323.7311.463 PMC1121065

[15] Khan MS, Coker RJ. How to hinder tuberculosis control: five easy steps. The Lancet. 2014;384:646–648. 10.1016/S0140-6736(14)61175-2 25064593

[16] Chuc NTK, Tomson G. “Doi moi” and private pharmacies: a case study on dispensing and financial issues in Hanoi, Vietnam. Eur J Clin Pharmacol. 1999;55:325–32. 10424327 10.1007/s002280050636

[17] Saw S, Lwin T, Khaing TMM, Myint B, Oo KS, Myint CC, et al Success and challenges of Public-Private Mix DOTS initiatives in Myanmar: a process evaluation for partnership approach of non-governmental organizations. Myanmar Health Sci Res J. 2009;21:186–93.

[18] Saw S, Mon M, Naing S, Mar K, Win W, Aye M. Existing practices of general practitioners on diagnosis and treatment of tuberculosis in Yangon. Myanmar Health Sci Res J. 2002;14:12–6.

